# Nasobiliary tube-assisted cholangioscopy-guided electrohydraulic lithotripsy successfully used to treat a difficult common bile duct stone

**DOI:** 10.1055/a-2760-9667

**Published:** 2026-01-16

**Authors:** Rengyun Xiang, Chaochao Chen, Jie Wang, Jiahui Tian, Xuefeng Li, Xia Peng

**Affiliations:** 1 74680 Department of Gastroenterology, The First Affiliated Hospital of Jishou University, Jishou, China; 2 74680 Department of Anesthesiology, The First Affiliated Hospital of Jishou University, Jishou, China; 3 480673 Jishou University School of Medicine, Jishou, China

Endoscopic retrograde cholangiopancreatography (ERCP) remains the most effective therapeutic modality for choledocholithiasis. However, for large common bile duct (CBD) stones, especially those with a diameter of >1.5 cm that are termed “difficult” CBD stones, additional interventional techniques are usually needed. Cholangioscopy-assisted electrohydraulic lithotripsy (EHL) is recommended as a safe and effective therapy for difficult CBD stones 1 2 . However, cholangioscopy-assisted EHL is challenging due to proximal migration and nonfixation of stones or fragments during the processing of CBD stones. Herein, we recommend a new method, nasobiliary tube-assisted cholangioscopy-guided EHL, that can significantly increase the efficiency and substantially reduce the difficulty of lithotripsy.

A 61-year-old woman was referred to our hospital for a difficult CBD stone, for which ERCP was unsuccessful at the previous hospital. Magnetic resonance imaging revealed an 18-mm stone in the CBD ( Fig. 1 ). Therefore, we planned to use nasobiliary tube-assisted cholangioscopy-guided EHL to crush the difficult stone ( Video 1 ). First, a nasobiliary tube (7 Fr) was inserted with its proximal end located above the stone, which functioned as a temporary fixation and antiretropulsive device ( Fig. 2 **a** ). EHL was subsequently performed under the direct visualisation of cholangioscopy ( Fig. 2 **b** ). During lithotripsy, the stone was anchored at the impacted site by the nasobiliary tube, with no fragment migrating in a retrograde manner into the upstream bile duct. Consequently, the stone was sufficiently fragmented, and the fragments were repeatedly removed using a 5-Fr basket ( Fig. 2 **c** ). Finally, cholangioscopy revealed no stones or fragments in the CBD ( Fig. 2 **d** ). The total procedure time was 32 minutes, and there were no adverse events.

Nasobiliary tube-assisted cholangioscopy-guided electrohydraulic lithotripsy successfully used to treat a difficult common bile duct stone.Video 1

**Fig. 1:**
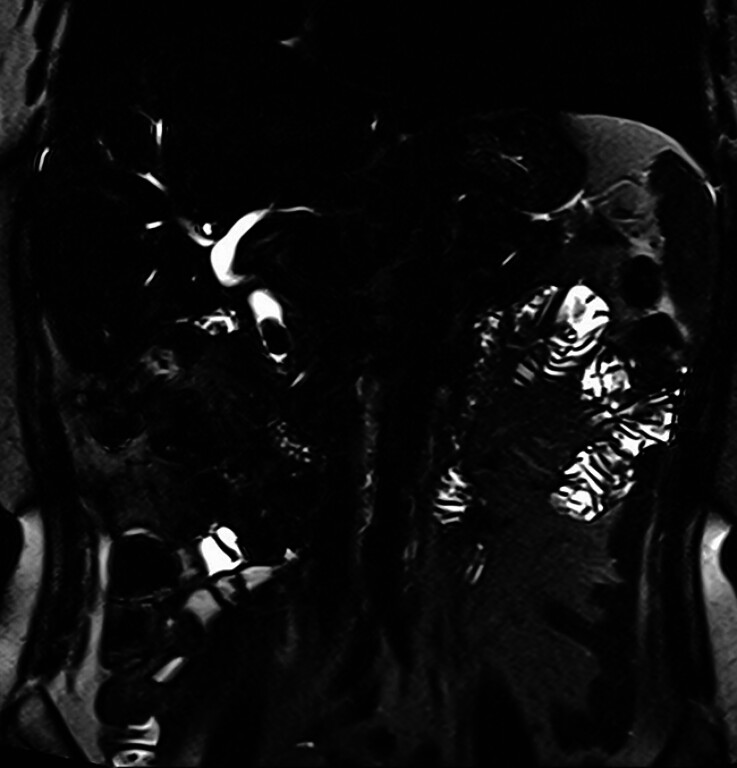
Magnetic resonance imaging revealed an 18-mm stone in the common bile duct.

**Fig. 2:**
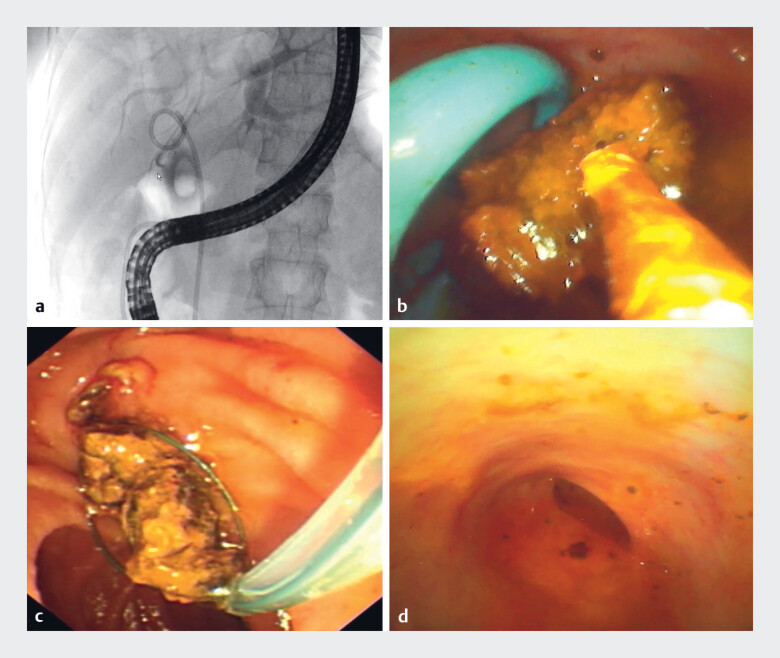
**a** A nasobiliary tube was inserted and the proximal end of it was located above the stone. **b** Electrohydraulic lithotripsy was subsequently performed under the direct visualisation of cholangioscopy. **c** Stone fragments were removed using a 5-Fr basket. **d** The cholangioscopy revealed no stones or fragments in the CBD.

In conclusion, nasobiliary tube-assisted cholangioscopy-guided EHL was safe and effective. The nasobiliary tube effectively stabilized the stone, thereby reducing the difficulty of lithotripsy. Additionally, it prevented the retrograde migration of fragments into the upstream bile duct, improving the stone clearance rate.

Endoscopy_UCTN_Code_CPL_1AK_2AF

## Notes

Correction **Correction: Nasobiliary tube-assisted cholangioscopy-guided electrohydraulic lithotripsy successfully used to treat a difficult common bile duct stone** Xiang Rengyun, Chen Chaochao, Wang Jie et al. Nasobiliary tube-assisted cholangioscopy-guided electrohydraulic lithotripsy successfully used to treat a difficult common bile duct stone. Endoscopy 2026; 58: E78–E79, doi:10.1055/a-2760-9667 In the above-mentioned article the page numbers have been corrected. This was corrected in the online version on January 21, 2026.

## References

[1] Manes G Paspatis G Aabakken L Endoscopic management of common bile duct stones: European Society of Gastrointestinal Endoscopy (ESGE) guideline Endoscopy 2019 51 472 491 30943551 10.1055/a-0862-0346

[2] Troncone E Mossa M De Vico P Difficult Biliary Stones: A Comprehensive Review of New and Old Lithotripsy Techniques Medicina (Kaunas) 2022 58 120 35056428 10.3390/medicina58010120 PMC8779004

