# Correction: Nasobiliary tube-assisted cholangioscopy-guided electrohydraulic lithotripsy successfully used to treat a difficult common bile duct stone

**DOI:** 10.1055/a-2792-3478

**Published:** 2026-01-22

**Authors:** Rengyun Xiang, Chaochao Chen, Jie Wang, Jiahui Tian, Xuefeng Li, Xia Peng

**Affiliations:** 1 74680 Department of Gastroenterology, The First Affiliated Hospital of Jishou University, Jishou, China; 2 74680 Department of Anesthesiology, The First Affiliated Hospital of Jishou University, Jishou, China; 3 480673 Jishou University School of Medicine, Jishou, China



In the above-mentioned article the page numbers have been corrected. This was corrected in the online version on January 21, 2026.

